# Increased autophagy in fibroblast-like synoviocytes leads to immune enhancement potential in rheumatoid arthritis

**DOI:** 10.18632/oncotarget.14331

**Published:** 2016-12-28

**Authors:** Ru Yang, Yingzi Zhang, Lin Wang, Ji Hu, Jian Wen, Leixi Xue, Mei Tang, Zhichun Liu, Jinxiang Fu

**Affiliations:** ^1^ Department of Rheumatology, The Second Affiliated Hospital to Soochow University, Jiangsu, China; ^2^ Department of Orthopaedics, The Second Affiliated Hospital to Soochow University, Jiangsu, China; ^3^ Department of Laboratory Medicine, The First Affiliated Hospital to Soochow University, Jiangsu, China; ^4^ Department of Endocrinology, The Second Affiliated Hospital to Soochow University, Jiangsu, China; ^5^ Department of Hematology, The Second Affiliated Hospital to Soochow University, Jiangsu, China

**Keywords:** rheumatoid arthritis, autophagy, fibroblast-like synoviocytes, IL-6

## Abstract

The incidence of rheumatoid arthritis (RA) has been reported to be correlated with a disorder of immunregulation. Rheumatoid arthritis fibroblast-like synoviocytes (RA-FLSs) play an important role in regulating the local immune microenvironment. However, the potential mechanism of RA-FLS in regulating the immnue response is not clearly understood. In this study, we demonstrated that the expression of HIF-1α was significantly up-regulated in rheumatoid arthritis tissue which indicated that the hypoxia condition in the microenvironment. We also observed that RA-FLSs demonstrated the potential to up-regulate immune activation. Meanwhile, the level of autophagy increased in RA-FLSs compared with control group. Besides that, the expression of IL-6 was up-regulated not only in RA-FLSs but also in the fibroblasts that treated with hypoxia condition. Accordingly, we found that autophagy inhibitiors could effectively inhibit the immune activation function of RA-FLSs medicated by IL-6. Taken together, the results we demonstrated above indicated that the hypoxia microenvironment could effectively induce the incidence of autophagy and then lead to the immune activation function of RA-FLSs medicated by IL-6.

## INTRODUCTION

Rheumatoid arthritis (RA) is an autoimmune-mediated chronic inflammatory joint disease which affects approximately 1% of the population with disability and decreased quality of life in the end-stage [1, 2]. Many investigations have indicated that RA is caused by genetic and/or environmental factors and characteristically affect the small joints of the hands and feet which often lead to joint structure destruction and functional impairment [3, 4]. Increased studies demonstrated the pathogenetic mechanism of RA was caused by cytokine networks and associated cells [5]. Many interventions have been employed in treating RA and gained a great progress in treating RA, like inhibiting interleukin (IL)-1, IL-6 and tumor necrosis factor (TNF)αsignaling pathway, or target pathogenic cells such as osteoclasts and B cells [6, 7]. However, two major problems still unresolved one is up to 30% of RA patients fail to respond to treatments, and the other is radiographic progression of joint damage even accompany with clinical remission of the inflammatory component. Consequently, inhibition of inflammation may not be sufficient to suppress the progression of RA and the mechanisms of RA need further exploring.

Fibroblast-like synoviocytes (FLS), located in the synovial tissue and strong correlation with the progress of RA, has drawn many researchers’ attentions [8, 9]. Meanwhile, FLS precursors has been demonstrated could migrate to the synovium via pores in cortical bone, as has been demonstrated in mice with collagen-induced arthritis [10]. Compared to healthy FLS, Rheumatoid arthritis fibroblast-like synoviocyte (RA-FLS) has acquired MHC class II and works as an antigen presenting cell which can cause T cell activation and proliferation in a comparable way to antigen present cells (APCs)[11]. In addition, the mechanisms of RA-FLS inducing the activation and proliferation of T cells by interacting between CD47 expressed on T cell surface and its ligand (thrombospondin-1) expressed by FLS or interacting between CXCR4 expressed on T cell surface and its ligand (stromal cell-derived factor-1, SDF-1) expressed by FLS [12]. Furthermore, RA-FLS can directly aggravate the inflammatory processes through enhancing to the local production of cytokines, inflammatory small molecule mediators and proteolytic enzymes that degrade the extracellular matrix [13]. Based on the understanding of RA-FLS biology, RA-FLS is described as a potential targeting cell in treating RA which might improve clinical outcomes in inflammatory arthritis without suppressing systemic immunity. However, despite many studies and methods were employed in inhibiting the progress of RA by suppressing RA-FLS, the prognosis remains unsatisfied. Thus, further study of the mechanism of RA-PLS is needed.

Former studies indicated that inflammatory-related tissue damage can lead to local blood supply deficiency which can form a hypoxic microenvironment [14]. It has been reported that hypoxic stress could effectively induce the incidence of autophagy in many cells [15–17]. However, the influence and internal mechanism of RA-FLS immune regulating function affected by hypoxic microenvironment is still unclear.

In our present study, we aim to observe the effect of RA-FLS immune regulating function influenced by hypoxic microenvironment and further explore the potential mechanism. The result demonstrated that hypoxic stress can obviously increase the autophagy of RA-PLSs and inhibition the autophagy can significantly down-regulated the immune activation function of RA-FLSs. Furthermore, we found that the mechanism of autophagy increased by hypoxic stress enhanced the immune activation function via up-regulated the expression of IL-6.

## RESULTS

### The expression of HIF-1α was significantly up-regulated in rheumatoid arthritis tissue

**Figure 1:**
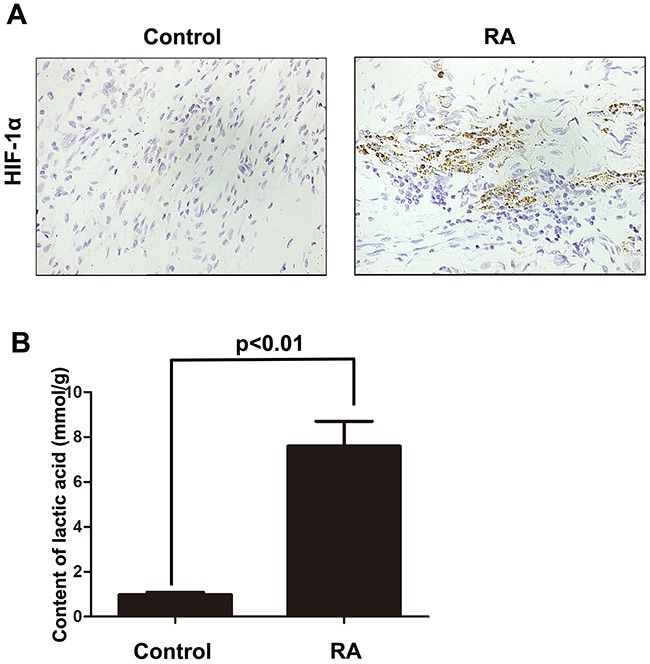
The expression of HIF-1α was significantly up-regulated in rheumatoid arthritis tissue **A**. IHC was employed to detect the expression of HIF-1α in rheumatoid arthritis tissues. **B**. Lactic acid detection assay was used to examine the hypoxic state in rheumatoid arthritis tissues. Data represent three independent experiments with SD. Statistically significant differences are shown (*P < 0.05; **P<0.01).

Firstly, we constructed the animal model of rheumatoid arthritis. In the tissues of rheumatoid arthritis, we could observe that the expression of HIF-1α was significantly up-regulated, which indicated that the hypoxia condition in the microenvironment (Figure 1A). Besides that, we also employed lactic acid detection assay to confirm the hypoxia microenvironment in rheumatoid arthritis tissues. As shown Figure 1B, we found that hypoxia microenvironment existed in rheumatoid arthritis tissues compared with normal control group. These results indicate that the incidence of rheumatoid arthritis may associate with hypoxia microenvironment.

### RA-FLSs demonstrated the potential to up-regulate immune activation

**Figure 2:**
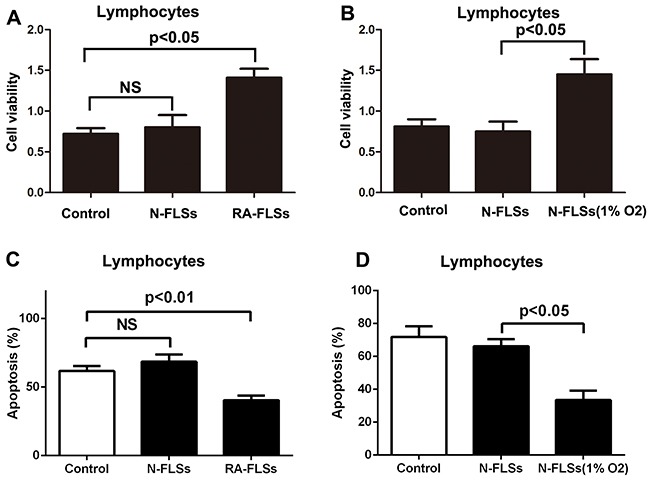
RA-FLSs demonstrated the potential to up-regulate immune activation **A-B**. CCK-8 assay was used to detect the cell viability of lymphocytes. **C-D**. PI/Annexin V-FITC assay was used to examine the apoptosis of the lymphocytes by flow cytometry. Data represent three independent experiments with SD. Statistically significant differences are shown (*P < 0.05; **P<0.01).

RA-FLSs were isolated from rheumatoid arthritis tissues. Then the effect of RA-FLSs on the cell viability and apoptosis of lymphocytes was detected through cultivated with supernate of RA-FLSs. As shown in Figure 2A, compared with control group, RA-FLSs could effectively enhance the cell viability of lymphocytes, whereas N-FLSs had no obvious effect on the proliferation of lymphocytes [18]. However, the FLSs collected from normal tissues were cultured in hypoxia condition, the N-FLSs then could significantly promote the cell viability of lymphocytes compared with control group (Figure 2B). Besides, we also observed that RA-FLSs decreased the apoptosis of lymphocytes and N-FLSs had no significant on the apoptosis. Furthermore, the N-FLSs pretreated with hypoxia condition could also decrease the apoptosis of lymphocytes (Figure 2C and 2D). These results implied that hypoxia condition may be a key factor in activating the potential of FLSs to up-regulate immune activation.

### The level of autophagy increased significantly in RA-FLSs

**Figure 3:**
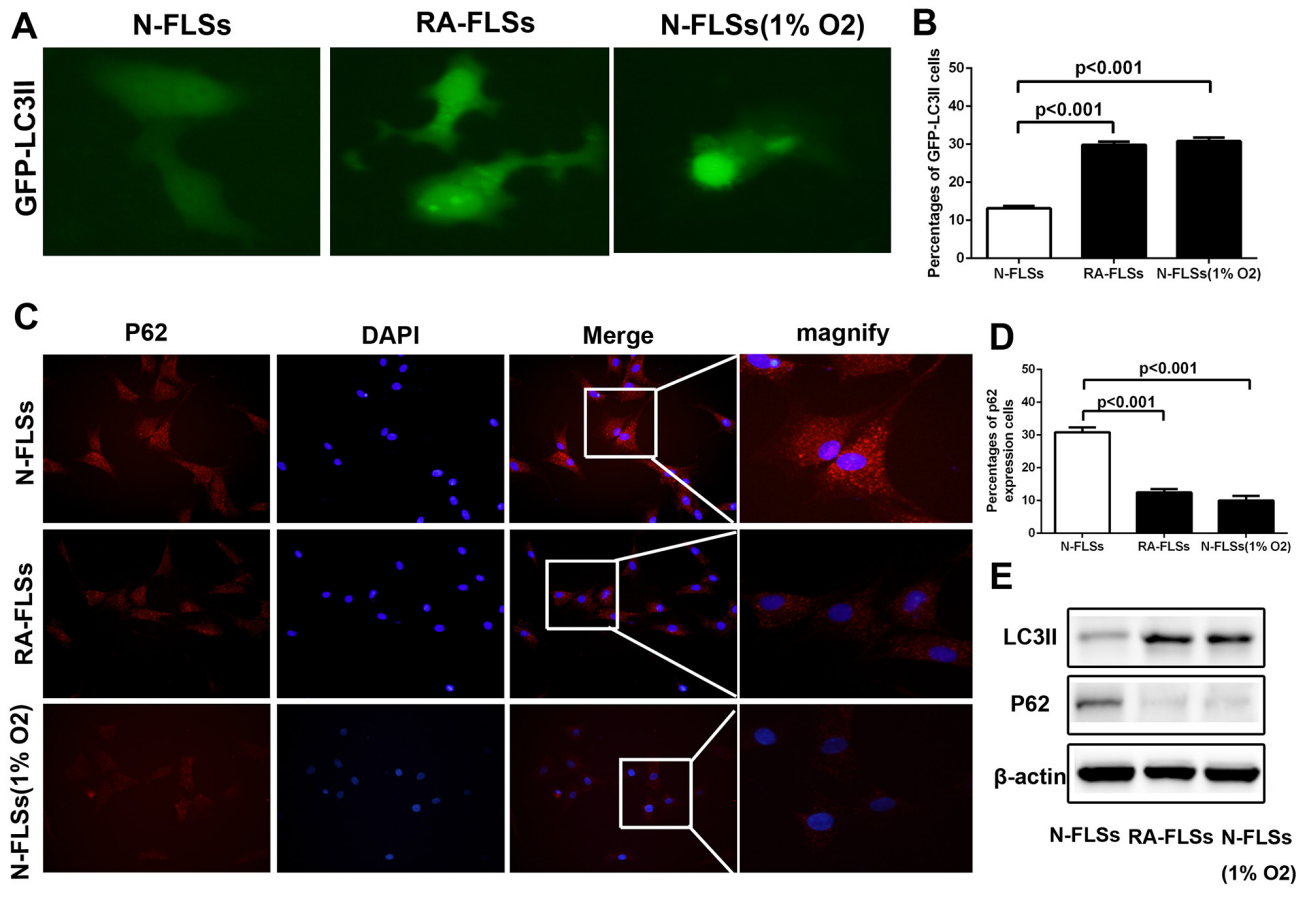
The level of autophagy increased significantly in RA-FLSs **A**. N-FLSs were transfected with GFP-tagged LC3; after 24 hours transfection, the N-FLSs cultured in hypoxia condition (1% O_2_) for 8 hours and then were observe by a fluorescence microscope. RA-FLSs were transfected with GFP-tagged LC3; after 24 hours transfection, the RA-FLSs were observed by a fluorescence microscope. Arrows show the punctate GFP-LC3 in the cytoplasm (×1000). **B**. The average percentages of GFP-LC3II cells. **C**. Immunfluorescence was employed to observe the expression of p62 in RA-FLSs and N-FLSs. N-FLSs were obtained from control rat model (×1000). **D**. The average percentages of GFP-LC3II cells. **E.** Western blot were used to examine the expression of LC-3II and p62.

It has been reported that hypoxic stress could effectively induce the incidence of autophagy [15–17]. Then we observed the level of autophagy in RA-FLSs and N-FLSs by using a GFP-LC3 expression vector which the primarily diffused fluorescence changed into a punctate fluorescence within cells undergoing autophagy. As shown in Figure 3A, the level of autophagy in RA-FLSs is significantly higher than N-FLSs, which exhibited a high number of punctate GFP. Meanwhile, hypoxia condition could effectively lead to the occurrence of autophagy in N-FLSs. Meanwhile, the average percentages of GFP-LC3II cells was counted and the result was consistent (Figure 3B). Besides that, we also employed immunofluorescence to observe p62 expression in N-FLSs, RA-FLSs and N-FLSs cultured in hypoxic condition. The results showed that the expression of p62 in RA-FLSs and N-FLSs cultured in hypoxic condition obviously decreased which indicated that high level of autophagy in RA-FLSs and the N-FLSs cultured in hypoxic condition than N-FLSs (Figure 3C). At the same time, the average percentages of p62 cells was also counted and the result was consistent (Figure 3D). The results from western blot also demonstrated that LC-3II expression was also up-regulated in RA-FLSs and N-FLSs that treated with hypoxia condition by contrasting with N-FLSs. And the p62 expression down-regulated in RA-FLSs and N-FLSs that treated with hypoxia condition by contrasting with N-FLSs (Figure 3E).

### Autophagy inhibitors could effectively inhibit the immune activation function of RA-FLSs

**Figure 4:**
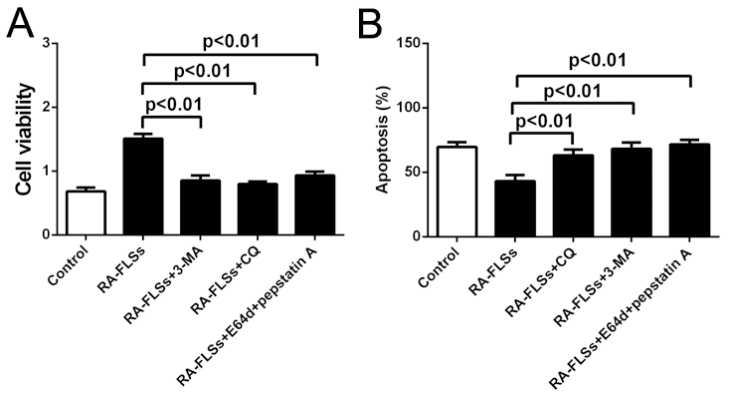
Autophagy inhibitors could effectively inhibit the immune activation function of RA-FLSs **A**. CCK-8 assay was used to detect the cell viability of lymphocytes **B**. PI/Annexin V-FITC assay was used to examine the apoptosis of the lymphocytes by flow cytometry with or without autophagy inhibitor 3-MA and CQ. Data represent three independent experiments with SD. Statistically significant differences are shown (*P < 0.05; **P<0.01).

In order to confirm the role autophagy in the immune activation function of RA-FLSs, we used autophagy inhibitor 3-methyladenine (3-MA) and Chloroquine (CQ) to inhibit the autophagy in RA-FLSs. The results showed that the enhancement of RA-FLSs on the cell viability of lymphocytes was significantly decreased by 3-MA and CQ (Figure 4A). Meanwhile, the apoptosis of lymphocytes increased when cocultured with RA-FLSs that the autophagy was inhibited by 3-MA and CQ compared with control group (Figure 4B). These results suggested that autophagy might play an important role in the immune activation function of RA-FLSs

### The expression of IL-6 was up-regulated not only in RA-FLSs but also in N-FLSs that treated with hypoxia condition

**Figure 5:**
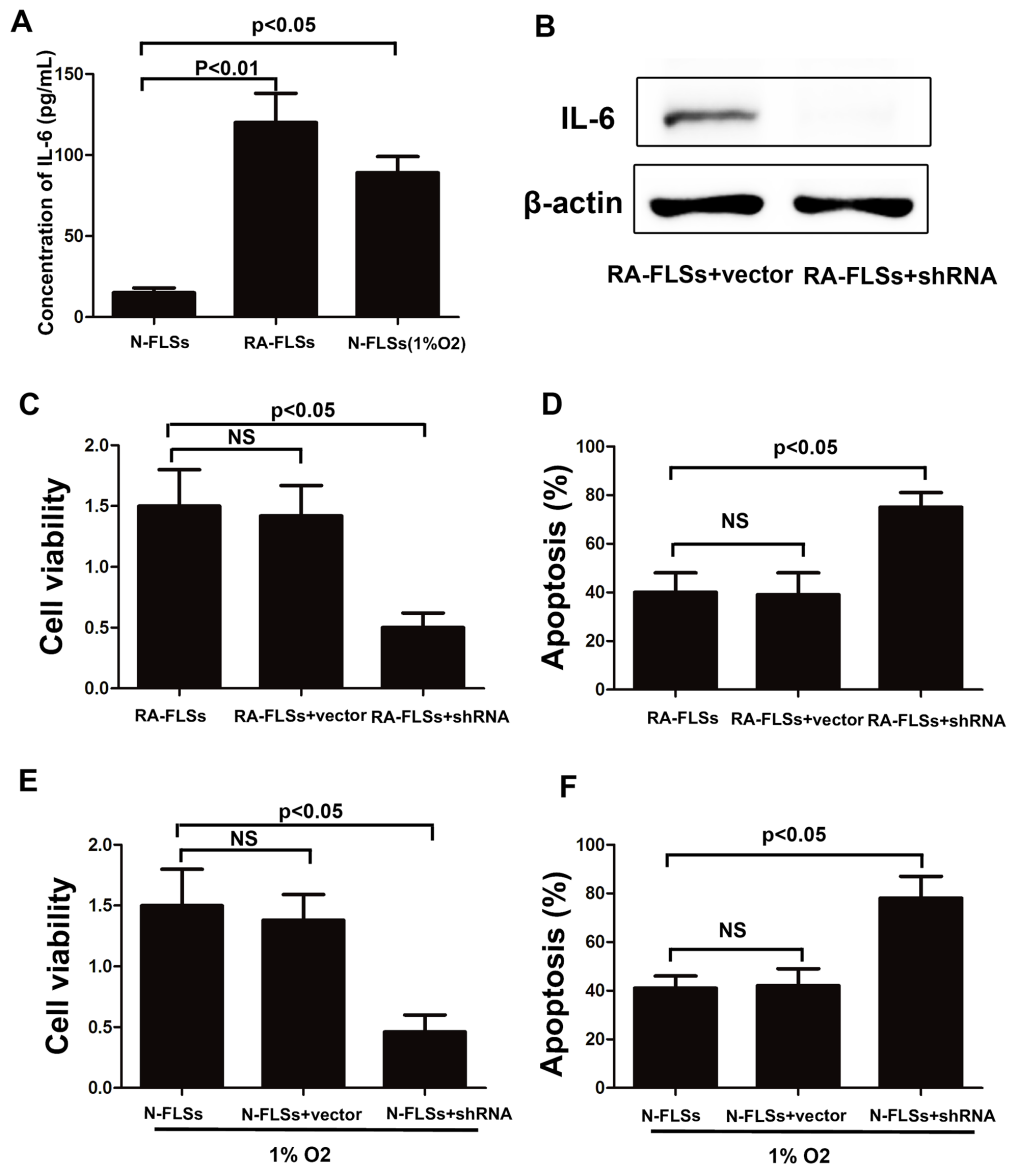
The expression of IL-6 was up-regulated not only in RA-FLSs but also in the fibroblasts that treated with hypoxia condition **A**. ELISA was employed to detect the expression of IL-6 in the conditioned mediums obtained from RA-FLSs and N-FLSs; **B** and **D**. CCK-8 assay was used to detect the cell viability of lymphocytes with or without inhibiting the expression of IL-6 by shRNA in RA-FLSs; **C** and **F**. PI/Annexin V-FITC assay was used to examine the apoptosis of the lymphocytes by flow cytometry with or without inhibiting the expression of IL-6 by shRNA in RA-FLSs. Data represent three independent experiments with SD. Statistically significant differences are shown (*P < 0.05; **P<0.01, NS means no significant).

It has been reported that IL-6 is a key immune factor which is up-regulated in the tissue of RA. Then we assess the secretion of IL-6 in RA-FLSs and N-FLSs by ELISA. As shown in Figure 5A, the expression of IL-6 was significantly higher in RA-FLSs compared with N-FLSs. Meanwhile, hypoxia condition could effectively induce the up-regulation of IL-6 in N-FLSs. Furthermore, blockade of IL-6 expression by shRNA in RA-FLSs diminished the ability of RA-FLSs in immune activation by cultivating lymphocytes with the supernate of RA-FLSs (Figure 5B and 5C). The result is also demonstrated in N-FLSs with hypoxia condition contrasted with N-FLSs (Figure 5D and 5E). Collectively, these results confirmed that IL-6 was the key factor that mediated the immune activation function of RA-FLSs and hypoxia condition might play an important role in up-regulate the expression of IL-6 in N-FLSs.

### Autophagy mediated the up-regulation of IL-6 in RA-FLSs

**Figure 6:**
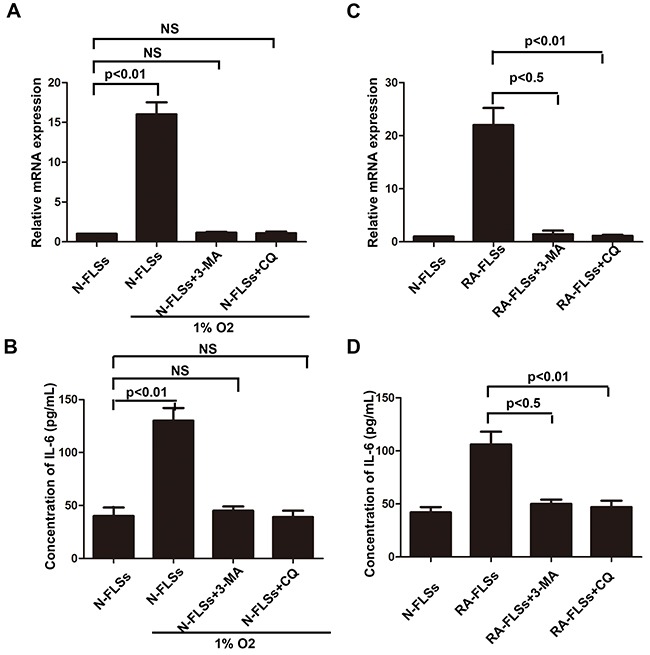
Autophagy mediated the up-regulation of IL-6 in RA-FLSs **A** and **C**. Realtime PCR was used to detect the expression of IL-6 in N-FLSs and RA-FLSs; **B** and **D**. ELISA was employed to detect the expression of IL-6 in the conditioned mediums obtained from RA-FLSs and N-FLSs Data represent three independent experiments with SD. Statistically significant differences are shown (*P < 0.05; **P<0.01, NS means no significant).

We have demonstrated that hypoxia condition could induce the incidence of autophagy, meanwhile it also lead to up-regulation of IL-6 in RA-FLSs. We employed autophagy inhibitor 3-MA and CQ to confirm the role of autophagy in up-regulating the expression of IL-6 not only in N-FLSs treated with hypoxia condition but also in RA-FLSs. The results demonstrated that when the autophagy was inhibited, hypoxia condition could not enhance the expression of IL-6 in N-FLSs (Figure 6A). As shown in Figure 6C and 6D, inhibition of autophagy by 3-MA and CQ could lead to the down-regulation of IL-6 in RA-FLSs. The result suggested that the IL-6 associated immune activation function of RA-FLSs induced by hypoxia microenvironment may be mediated by autophagy.

## DISCUSSION

In our study, we showed the effect of immune activation function influenced by hypoxic condition in RA-FLSs and explored the possible mechanisms. Firstly, we found the high expression of HIF-1α by IHC in the animal with rheumatoid arthritis which indicated the incidence of rheumatoid arthritis is associated with hypoxia microenvironment. Subsequently, we separated the RA-FLS and healthy FLS from rheumatoid arthritis tissues and normal and then treated to T cells by co-culture. By detecting the cell viability and apoptosis of lymphocytes, we found that RA-FLS could obviously enhance the cell viability and decreased the apoptosis by comparing to healthy FLS-treated. Moreover, compared to control group, the autophagy of RA-PLS was significantly up-regulated by analyzing the expression of LC3 and p62 which was cultured in hypoxic condition. To further demonstrated the relationship between autophagy and immune activation function, autophagy inhibitors (3-MA and CQ) were used and the results indicated that inhibition of autophagy often accompany with down-regulation of immune activation function in RA-FLS. Furthermore, ELISA was performed and we observed that the secretion of IL-6 in RA-FLSs treated with hypoxic condition was obviously up-regulated. Additionally, inhibition of autophagy by 3-MA and CQ could effectively block the up-regulation of IL-6 caused by hypoxia condition, which demonstrated that RA-FLS enhanced immune activation function in hypoxic condition via increased autophagy-mediate the expression of IL-6.

Rheumatoid arthritis is a chronic inflammatory and complex autoimmune disease [19]. Nowadays, many methods have been adopted to treat it [20–22]. Through it has got huge progress, yet up to 30% of RA patients are failed to respond to treatments, and many of them still have joint damage in radiographic progression even accompanied with clinical remission of the inflammatory component. All of above indicated that inhibition the function of inflammatory cytokines, inflammatory cells or damaged-targeting osteoclasts cannot cure RA efficiently [23]. With the progression of studies, FLSs has drawn many researcher's attentions which has been thought as a potential targeting cell in treating RA [8]. Many studies about the function and biology of FLS have been explored, while the effect on interfering FLS is still unsatisfied.

Former studies indicated that inflammatory-related tissue damage can lead to local blood supply deficiency which can form a hypoxic microenvironment [24, 25]. Hypoxic stress, marked as the high expression of hypoxia-inducible factor-1α(HIF-1α), vascular endothelial growth factor (VEGF) and carbonic anhydrase IX (CAIX), could induce drug resistance and down-regulate the therapeutic effect [26]. Especially, the HIF-1αhas been demonstrated taking a relevant role in drug-resistance [27]. In our study, we also demonstrated that the expression of HIF-1α, which is a marker of hypoxic stress, is significantly increased in the animal with rheumatoid arthritis (Figure 1A and 1B). In addition, the immune activation of RA-FLSs is obviously higher than healthy FLSs (Figure 2A and 2B). To further explore the mechanism, the LC3 and P62 were analyzed and the result indicated that hypoxic can effectively induce the incidence of autophagy in RA-FLSs. Importantly, we found that the high incidence of autophagy in RA-FLSs which can enhance the immune activation function by increased in secretion of IL-6. Former studies have demonstrated that IL-6 can up-regulated the immune function by promote the proliferation of T cells. Present result also showed that inhibiting the autophage can suppress the secretion of IL-6. In conclusion, our study indicated that ameliorate the hypoxic environment or inhibit the autophagy may be an effective in treating RA and increase the cure rate and improve prognosis.

## MATERIALS AND REAGENTS

### Cell lines and reagents

The FLS cells were primary isolated from synovial tissues and cultured in Dulbecco's modified Eagle's medium (DMEM) (GIBCO, Invitrogen) with 10% fetal bovine serum (FBS) at 37°C in a humidified atmosphere containing 5% CO_2_. The 1% O_2_ condition was used as hypoxia condition. Chloroquine (CQ, Cat.C6628) and 3-methyladenine (3-MA, Cat.M9281) were purchased from Sigma-Aldrich.

### Animal model

All procedures involving animals were performed in accordance with the institutional animal welfare guidelines of Tongji University. Subcutaneous implantation of RA-FLS cells was performed in armpit areas of nude mice. Mice were examined three times per week.

## MATERIALS AND METHODS

### Assessment of cell viability and apoptosis

Cell viability was assessed by Cell Counting Kit-8 (CCK-8) assay. The lymphocytes were seeded in 96-well plates (5000 cells per well). After 72h culturing, CCK-8 (10μl) was added in each well. The plate was then incubated at 37°C for another 2 hours. The optical density (OD) value at 450nm of each well was examined by spectrophotometer. All determinations were carried out in sextuplicate. The lymphocytes (2×10^5^cells/well) were cultured in 6-well plate. According to the manufacturer's instruction (Keygen Biotech. Co., Ltd, Nanjing, China), PI/Annexin V-FITC assay was used to examine the apoptosis of the cells by flow cytometry. The lymphocytes were collected and washed with ice cold PBS three times. Then the cells were incubated in 300 μL of 1×binding buffer containing 5 μL PI and 5 μL Annexin V in dark at room temperature for 30 min. After incubation, the apoptosis of the cells was detected by BD FACS Calibur flow cytometer (BD FACS Calibur). The double positive cells (PI and Annexin V positive) were concerned as apoptotic cells [18]. And the results are expressed as the percentage of apoptotic cells.

### Lactic acid detection assay

Firstly, same weight of Joint soft tissues was obtained from nude mice and triturated to get the tissue homogenate after added same volume. Subsequently, the supernate was separated after centrifuging. Then we added same volume supernate, substrate buffer and coenzyme, and cultivated in 37°C, 15min after centrifuging. After that, we added 2,4-dinitrophenylhydrazine (DNPH) and cultivated in 37°C, 15min after centrifuging. Sodium hydroxide (NaOH) then was added and strewed 3min for detecting the OD values in 440nm wave. The content of lactic acid was calculated according to the stand samples. The procedures were performed according to the manuscript of lactic acid testing kit (A020-2, Nanjing jiancheng, Nanjing, China).

### Real-time RT-PCR

Total mRNA was isolated from synovial tissues or the cells by using Trizol reagent according to the protocol offered by the manufacturer. The expression of genes was determined by real-time PCR using the SYBR Green Master Mix Kit. The sequences of the primers were listed in Table 1. Thermocycler conditions included an initial hold at 50°C for 2 minutes and then 95°C for 10 minutes, which was followed by a two-step PCR program of 95°C for 15 seconds and 60°C for 60 seconds repeated for 40 cycles by using a Mx3000P QPCR System (Stratagene, USA). The amount of endogenous β-actin mRNA was used for an internal control for qPCR in each sample. Alteration of mRNA expression is presented as the fold change relative to an untreated control. Primer sequences are listed in Table as follows: IL-6 forward 5’-CCGGAGAGGAGACTTCACAG-3’ reverse5’-ACAGTGCATCATCGCTGTTC-3’, β–actin forward 5’-GCCAACACAGTGCTGTCTG-3’ reverse 5’-TGATCCACATCTGCTGGAAGG-3’.

**Table 1:** Sequence of the oligonucleotides for real-time PCR

Gene		Sequence (5’ to 3’)
β-actin	F	GCCAACACAGTGCTGTCTG
	R	TGATCCACATCTGCTGGAAGG
IL-6	F	CCGGAGAGGAGACTTCACAG
	R	ACAGTGCATCATCGCTGTTC

### Western blotting

All treated cells were washed with PBS and lysed by RIPA and PMSF at a ratio of 100:1 to obtain the total protein for Western blot. Equal amount of proteins was separated by SDS-PAGE and transferred to polyvinylidene fluoride membrane. After transferring, the polyvinylidene fluoride membrane was blocked in 5% fat-free milk/1 × TBS/0.1% Tween-20 for 1 h at room temperature and then incubated with primary antibodies with gentle agitation overnight at 4°C. And then, the membrane was washed with 1 × TBS/0.1% Tween-20 before incubated with the secondary antibody according to the species of primary antibody for 1 h at room temperature. Immunoblots were performed by using the BeyoECL Plus substrate system (Beyotime), followed by washing with 1 × TBS/0.1% Tween-20. Western blot analyzed of LC3A/B (Abcam ab128025) and GAPDH (AP0063, Bioworld).

### Short hairpin RNA (shRNA) synthesis and transfection

The shRNA sequences of genes were designed by using Oligoengine software and verified by nucleotide BLAST searches. According to the protocol from the manufacturer, the cells were plated in 6-well plates overnight and then Fugene HD transfection reagent (Roche) were used to transfect GFP-shRNA expression plasmids into the cells. A scrambled sequence was used as a negative control. At the end of each experiment, cells were observed by fluorescence microscope and/or Western Blot to determine transfection efficiency. The experiments were conducted in triplicate.

### Immunohistochemistry

The paraffin-embedded tissues were cut into 4-μm-thick slides and were deparaffinized and rehydrated. The slides were blocked with 10% normal goat serum, then rinsed in PBS and incubated for with affinity-purified antibody (HIF-1α) in 2% normal goat serum in PBS for overnight at 4°C. Then the primary antibody was rinsed by PBS, biotinylated secondary antibody (1:500) was added on the slides and incubated for 30 min. The second antibody was omitted and bound secondary antibody was detected by the 3,3’-diaminobenzidine (DAKO) chromogen by standard methods.

### Immunofluorescence

The cells were fixed in 4% paraformaldehyde and permeabilized with 0.1% Triton X-100 at room temperature for 15 min. Then the cells were incubated with primary antibody at 4°C overnight. Then the cells were washed with PBS and treated with secondary antibody at 37°C for 60 min. After PBS washing, the cells were stained with PADI to identify the nuclear. Then the cells were observed by a fluorescence microscope (Olympus IX70).

### Enzyme linked immunosorbent assay (ELISA)

A commercial ELISA kit (R&D Systems, Minneapolis, MN) were used to identify the expression of IL-6 in conditioned medium collected from FLSs. The assay was performed accordingly to the protocol provided by the manufacturer. Means and standard deviations of concentrations in triplicate samples were compared by t-test.

### Statistical analysis

All data, expressed as mean ± SEM, were from at least three separate experiments. Data sets were analyzed by SPSS 18.0 software (SPSS, Inc., Chicago, USA). Comparison was done with t test (unpaired or paired). All p values presented were two-sided. P<0.05 was considered to be statistically significant.
