# Correction: Increased autophagy in fibroblast-like synoviocytes leads to immune enhancement potential in rheumatoid arthritis

**DOI:** 10.18632/oncotarget.20371

**Published:** 2017-08-21

**Authors:** Ru Yang, Yingzi Zhang, Lin Wang, Ji Hu, Jian Wen, Leixi Xue, Mei Tang, Zhichun Liu, Jinxiang Fu

Copyright: Yang et al. This is an open-access article distributed under the terms of the Creative Commons Attribution License 3.0 (CC BY 3.0), which permits unrestricted use, distribution, and reproduction in any medium, provided the original author and source are credited.

**Present:** The affiliation information is incorrect.

**Correct:** The proper affiliations appear below.

Original article: Oncotarget. 2017; 8:15420-15430. https://doi.org/10.18632/oncotarget.14331

**Ru Yang**^1,*^, **Yingzi Zhang**^2,*^, **Lin Wang**^3,*^, **Ji Hu**^4^, **Jian Wen**^1^, **Leixi Xue**^1^, **Mei Tang**^1^, **Zhichun Liu**^1^, **Jinxiang Fu**^5^

^1^ Department of Rheumatology, The Second Affiliated Hospital of Soochow University, Jiangsu, China

^2^ Department of Orthopaedics, The Second Affiliated Hospital of Soochow University, Jiangsu, China

^3^ Department of Laboratory Medicine, The First Affiliated Hospital of Soochow University, Jiangsu, China

^4^ Department of Endocrinology, The Second Affiliated Hospital of Soochow University, Jiangsu, China

^5^ Department of Hematology, The Second Affiliated Hospital of Soochow University, Jiangsu, China

